# Effect of Surface States on Terahertz Emission from the Bi_2_Se_3_ Surface

**DOI:** 10.1038/srep10308

**Published:** 2015-05-19

**Authors:** Li-Guo Zhu, Brian Kubera, Kin Fai Mak, Jie Shan

**Affiliations:** 1 Institute of Fluid Physics & Terahertz Research Center, China Academy of Engineering Physics, Mianyang, Sichuan 621900, China; 2 Department of Physics, Case Western Reserve University, Cleveland, Ohio 44106, United States; 3 Department of Physics, The Pennsylvania State University, University Park, Pennsylvania 16802, United States

## Abstract

Three-dimensional topological insulators are materials that behave as an insulator in the interior, but as a metal on the surface with Dirac surface states protected by the topological properties of the bulk wavefunctions. The newly discovered second surface state, located about 1.5 eV above the conduction band in Bi_2_Se_3_ allows direct photoexcitation of the surface electrons in n-doped samples with a Ti:sapphire femtosecond laser. We have observed efficient THz generation from the Bi_2_Se_3_ basal plane upon femtosecond optical excitation. By performing polarization-resolved studies on the emitted THz spectrum, two emission mechanisms have been identified, namely, emission generated from the transient photocurrent under the influence of the surface depletion field and from nonlinear optical rectification. The two types of emission are governed by distinct selection rules. And while the former is characterized by a narrow-band spectrum, the latter, involving almost instantaneous optical transitions, has a broad bandwidth and is enhanced by the presence of resonant transitions. These two emission mechanisms are further separated by their distinct doping dependence upon exposure to ambient air. With surface selectivity, THz emission spectroscopy thus provides a valuable spectroscopic tool for studies of the optical conductivity and dynamics of the surface state in centrosymmetric Bi_2_Se_3_.

Three-dimensional topological insulators (TIs), characterized by an insulating bulk and a conducting surface electronic structure, have attracted tremendous interest in recent years^1^,^2^,^3^,^4^,^5^. Due to the inverted orbitals of the conduction and valence bands under strong spin-orbit interactions, a linear crossing of the electronic states at the TI surface is guaranteed by the bulk-boundary correspondence and is protected from imperfections in the samples^2^. The surface state possesses a relativistic massless dispersion with chiral spin texture^3^ and has been observed by the angle-resolved photoemission spectroscopy (ARPES)^3^,^4^,^5^. Many novel phenomena associated with excitation of the surface state have been predicted including quantized magneto-optical rotation^6^, surface spin transport^7^,^8^,^9^, and bulk topological phase transitions^10^,^11^. The response of as-grown samples of 3D TIs such as Bi_2_Se_3_ and Bi_2_Te_3_, however, is often dominated by their bulk properties due to the large doping densities and the pinning of the bulk chemical potential within the conduction band. This makes the study of the Dirac surface state very challenging. With recent advances in the growth of thin films of 3D TIs by molecular beam epitaxy (MBE), the bulk response has been significantly reduced leading to interesting transport^12^,^13^,^14^ and optical results^15^,^16^,^17^,^18^,^19^. Meanwhile, surface specific techniques such as second harmonic generation (SHG)^20^,^21^ have been employed to enhance the probe sensitivity to the surface state. Furthermore, a second unoccupied Dirac surface state, located 1.5 eV above the lowest conduction band, has been revealed by two-photon ARPES^22^. This state with similar properties and origin as the low-energy surface state opens up new opportunities for optical studies of the Dirac surface states in TIs.

In this work, we employ the terahertz (THz) emission spectroscopy based on femtosecond optical excitation to investigate the second surface state in Bi_2_Se_3_. The technique has been broadly utilized in the study of ultrafast carrier and spin dynamics in a variety of materials including semiconductors^23^, metals^24^, graphene^25^, and TIs^26^. One important mechanism of THz emission is optical rectification of the femtosecond excitation pulse, which is, similar to the SHG process, a second-order nonlinear process. In Bi_2_Se_3_ with inversion symmetry in the bulk, the second-order nonlinearity exists only at the surfaces where inversion symmetry is broken and is thus surface specific. As schematically demonstrated in Fig. 1a Ti:sapphire laser pulse centered around 1.5 eV directly couples the lowest-energy conduction band with the second Dirac surface state. In contrast to the SHG with detection at the ultraviolet frequencies, the emission at the THz or far-infrared frequencies is in complete resonance with the transitions between the surface electronic states and is thus expected to be much enhanced and carry rich information on the optical conductivity of the surface states.

Efficient THz emission has been detected from the basal plane of Bi_2_Se_3_ at room temperature in our experiment. By spectrally resolving the emitted THz radiation under different excitation and emission polarizations, we have identified emission originated from the optical rectification process. Strong THz emission has also been observed from transient photocurrents under the influence of a surface depletion field near the TI surface, which bears the signature of the bulk electronic properties. These two THz contributions have been verified by varying the doping density (likely due to Se reaction with water vapor^21^,^27^) in Bi_2_Se_3_ by exposing the samples to ambient air. THz emission from transient photocurrents in the surface depletion field is strongly dependent on doping, while the resonance enhanced emission from optical rectification has a weak dependence on doping. Our results indicate that THz emission spectroscopy can potentially provide a powerful non-contact probe of the dynamics of Dirac electrons in TIs and future studies of THz emission with variable excitation photon energy and controlled doping in TIs are warranted.

## Results

THz emission is observed from highly oriented layered Bi_2_Se_3_ (111) surfaces (basal plane) under excitation of femtosecond optical pulses of beam diameter ~1 mm. The optical pulses are centered around 800 nm, of 50 fs in pulse duration and 1 kHz in repetition rate. (See Method for more details.) The excitation pulse impinges on the samples at 45° and the emitted THz pulse is peaked around the reflection direction (Fig. 2).

Figure 3 shows the typical THz electric-field waveforms E(t) emitted from aged samples (>60 minutes in ambient air after cleavage). (See below for results on freshly cleaved samples.) Under p-polarized excitation (Fig. 3a), only p-polarized emission is observed while the s-polarized component is negligible (P_in_-P_out_ ≠ 0, P_in_-S_out_ 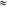 0). Similar result is observed for s-polarized excitation, i.e. S_in_-P_out_ ≠ 0, S_in_-S_out_ 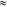 0. Under circularly polarized excitation, both s- and p-polarized emission are present with the s-component about 10 times weaker than the p-component in the electric-field amplitude and the two components nearly out of phase. Similar results are seen for both left and right circularly polarized excitation. Figure. 3b shows the case for left circularly polarized excitation: L_in_-P_out_ ≠ 0, L_in_-S_out_≠0. The THz amplitude spectra |E(Ω)| calculated through Fourier transform of the electric-field waveforms are shown in Fig. 3c,d for p-polarized and left circularly polarized excitation, respectively. A careful examination of these spectra also shows that while the p-polarized THz emission from aged samples for linearly and circularly polarized excitation has similar spectrum (peaked around 1 THz), the s-polarized emission displays a broader spectrum centered around 1.5 THz. We note that the latter spectral bandwidth is limited by that of our THz detection setup, which was calibrated using a 1 mm thick ZnTe emitter, a broadband emitter based on optical rectification.

Bi_2_Se_3_ samples were rotated about their surface normal to investigate the dependence of THz emission on the azimuthal orientation. For all the polarization combinations we did not observe any dependence of the emission on the azimuthal angle within the experimental uncertainty.

The evolution of the THz emission was recorded in real time after freshly cleaved Bi_2_Se_3_ samples were exposed to ambient air. At time zero, samples were cleaved and kept in a chamber purged by dry air gas. Dry air purging was stopped after 30 minutes. THz emission was recorded for about 2 hours for various polarization combinations. Figure 4 and 5 show the result for p-polarized excitation and p-polarized emission (P_in_-P_out_). In contrast, the emission from freshly cleaved samples (Fig. 4a) is much weaker than that from aged samples (Fig. 3a) and has a broader emission spectrum (Fig. 4d), similar to that of the L_in_-S_out_ emission from aged samples (Fig. 3d). After exposure to ambient air (indicated by the red arrow in Fig. 5a), the THz emission quickly increases over a time duration of about 20 minutes and remains constant upon further exposure to air. The THz electric-field waveform and amplitude spectrum at time B and C (shortly after exposure to air and 60 min. after exposure) are shown in Fig. 4b,c,e,f, respectively. Upon exposure to air, the low frequency emission amplitude increases while the high frequency components remain largely unaffected. This trend is summarized in Fig. 5c for the emitted THz intensity |E(Ω)| at two representative frequencies 1 THz (purple) and 2 THz (red). Furthermore, to exclude the influence of laser irradiation, in Fig. 5b we show the same sequence as in Fig. 5a except that the emission was not measured for the first 60 min. and the sample was not exposed to the laser beam. Finally, p-polarized THz emission under circularly polarized excitation (L_in_-P_out_) shows similar temporal and spectral evolution as that of P_in_-P_out_, while P_in_-S_out_ THz emission remains zero and L_in_-S_out_ THz emission keeps almost constant.

## Discussion

**Figure 1:**
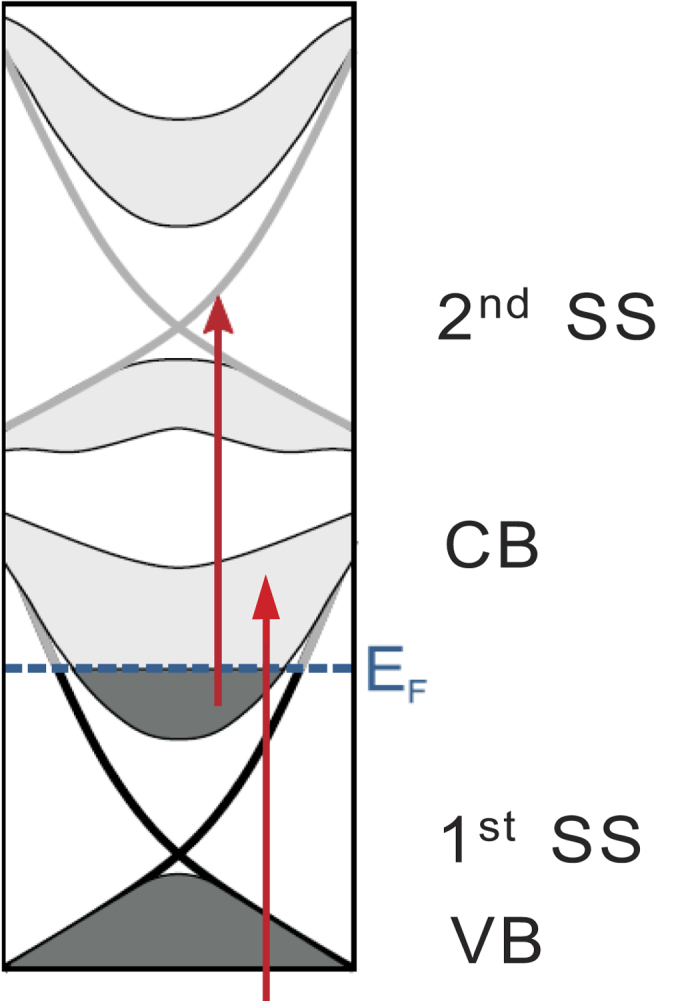
Schematic illustration of the optical excitation processes in (111) surface of n-doped Bi_2_Se_3_ by a ~1.5 eV photon (modified from Ref. 22). Surface electrons are directly excited by optical transitions from the conduction band (CB) to the second surface state (SS). Bulk electrons are directly excited by optical transitions from the valence band (VB) to the CB.

**Figure 2:**
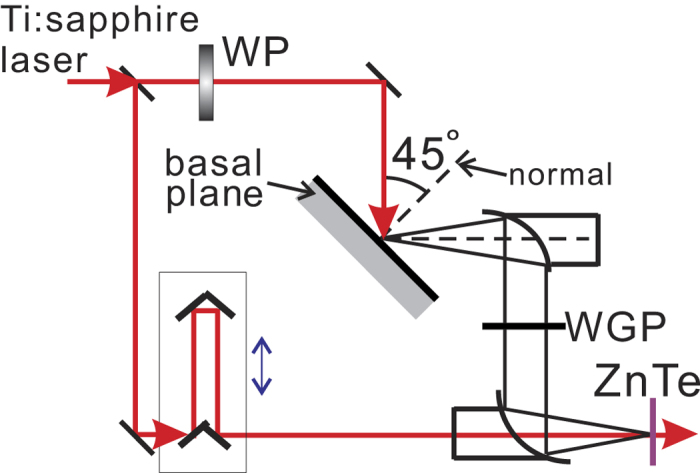
Schematic diagram of the experimental setup of THz emission spectroscopy. The Ti:sapphire laser beam is divided into two parts. The transmitted part of a chosen polarization by a wave-plate (WP) impinges on the sample surface at 45°. The emission after filtering the excitation beam is collected by a parabolic mirror in the reflection geometry and focused onto a ZnTe crystal. The polarization of the THz radiation is selected by a wire grid polarizer (WGP) placed between the two parabolic mirrors. The reflected laser beam passes through a delay line and is used for detection of the THz electric-field waveform in the ZnTe crystal through electro-optical sampling.

**Figure 3:**
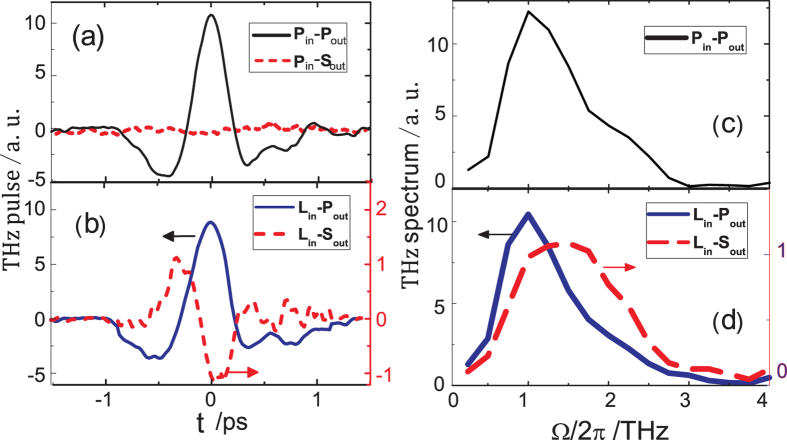
Selection rules of THz emission from aged Bi_2_Se_3_ samples under ultrafast laser excitation. (**a**) and (**b**) are the s- and p-polarized electric-field waveforms of THz emission E(t) under p-polarized and left circularly polarized excitation, respectively. (**c**) and (**d**) are the corresponding THz amplitude spectra |E(Ω)|.

**Figure 4:**
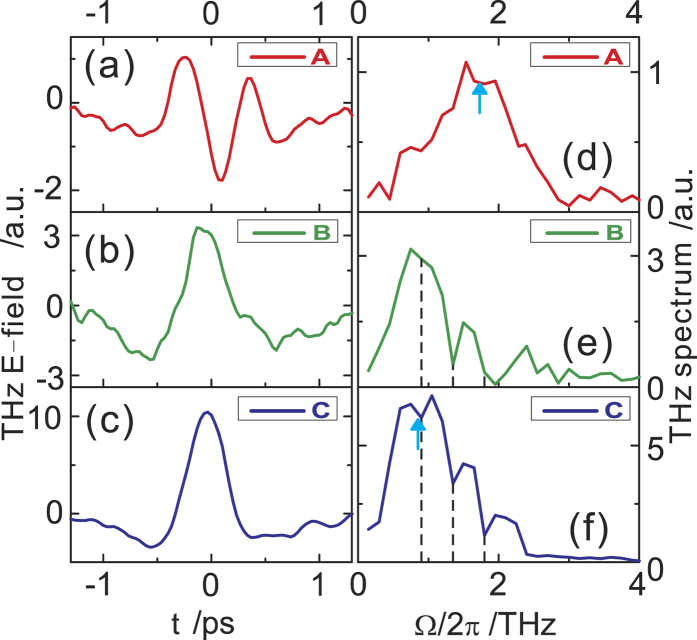
Evolution of the p-polarized THz emission under p-polarized excitation after cleavage of Bi_2_Se_3_ sample surfaces. (**a**)–(**c**) represent the THz electric-field waveforms and (**d**)–(**f**) are the corresponding amplitude spectra. Waveform A was measured on a fresh sample in a dry air purged chamber; waveform B was measured shortly after purging was stopped and waveform C about 1 hour later. Time A, B, C are also marked in Fig. 5(a) in the time dependence of the emission intensity. The dashed lines in (**e**) and (**f**) indicate strong water vapor absorption lines within the THz spectrum.

Several mechanisms can give rise to THz emission from a semiconductor surface under femtosecond optical excitation above the material’s band gap. They include the photo-Dember effect^23^, the transient photocurrent under the surface depletion or accumulation field^23^,^28^, and optical rectification^29^,^30^,^31^,^32^. In highly oriented layered Bi_2_Se_3_ samples which are typically n-doped due to Se vacancies, the optical pulse directly excites surface electrons (from the conduction band to the second surface state) and bulk carriers (primarily from the valence band to the conduction band) (Fig. 1). The latter density is expected to dominate. THz generation through the photo-Dember effect due to the different electron and hole mobility in the out-of-plane direction is negligible in Bi_2_Se_3_ because of the similar electron and hole mobilities^33^. THz emission can be generated by transient photocurrents in the surface deletion region, where a surface field *E*_*surf*_ formed by band bending due to doping accelerates the photo-injected carriers along the surface normal direction. The transient photocurrent along the surface normal, however, can only emit p-polarized THz radiation and cannot account for the s-polarized emission observed in our experiment.

Optical rectification in Bi_2_Se_3_ can be described by the nonlinear polarization at the THz frequencies Ω as 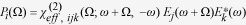. Here *E* and *ω* are the electric field and frequency of the excitation beam, respectively; the subscripts *i, j* and *k* denote the polarization of the THz and the excitation field; the effective second-order nonlinear susceptibility is composed of the second-order nonlinear susceptibility *χ*^*(2)*^and a dc field induced effect through the third-order nonlinear susceptibility *χ*^*(3)*^ and the surface field: 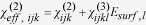. As discussed above, *χ*^*(2)*^ vanishes in bulk Bi_2_Se_3_ since the material is centrosymmetric. It is nonzero only within a few atomic layers of the sample surface, where inversion symmetry is broken. The *χ*^*(3)*^ contribution, being nonzero for any crystallographic groups, is usually weaker under typical surface fields *E*_*surf*_. 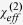 and the emission can be greatly enhanced if *ω* and/or Ω are in resonance with any electronic transitions. We adopt the result (and notations) derived for the SHG in Bi_2_Se_3_^20^,^34^ and express the THz polarization under p-polarized and left circularly polarized excitation in Eqn. 1. The results for s-polarized and right circularly polarized excitation are similar and are omitted here.


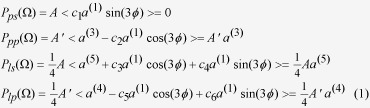


Here the first subscript for THz polarization *P* denotes the polarization state of the excitation and the second, the polarization state of the emission. The scaling factors *A* and *A’* contain the local field correction factors and the instrumental collection efficiencies for the emitted THz radiation. *c*_*k*_ are constants. *a*^*(k)*^ (*k* = 1 – 6) are the components of the effective nonlinear susceptibility with local field correction factors for the excitation field. And *ϕ* denotes the sample azimuthal orientation. The Bi_2_Se_3_ samples investigated in this experiment are not single crystals. The samples consist of many crystalline domains within the excitation area. Since the different domains of micro-crystallites are randomly oriented in the basal plane but well ordered in the <111> direction, we perform an average over the azimuthal angle *ϕ*. The ensemble averaged THz emission is independent of azimuthal angle *ϕ*, consistent with the experiment. In addition, the s-polarized emission vanishes under s- or p-polarized excitation.

Combining the polarization selection rules for two emission mechanisms, we conclude that the THz emission for P/S_in_-S_out_ 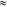 0 since neither transient photocurrent nor optical rectification gives rise to nonzero emission; and the nonzero emission P/S_in_-P_out_ ≠ 0 and L/R_in_-P_out_ ≠ 0 include contributions from both mechanisms; L/R_in_-S_out_ ≠ 0 arises solely from optical rectification and is therefore a good probe of the surface electrons. The assignment is fully compatible with the observed polarization selection rules for the THz emission. It is also compatible with the observed difference in the bandwidth of the emission. Contribution from the transient photocurrent involves bulk carriers transporting in the out-of-plane direction and the emission bandwidth is limited by the momentum relaxation and the carrier recombination rates. On the other hand, the contribution from optical rectification involves the rapid relaxation of the highly excited electronic states and can give rise to a broader bandwidth. The observed p-polarized emission from freshly cleaved samples is thus likely dominated by the surface electrons, and in aged samples, by the bulk electrons.

**Figure 5:**
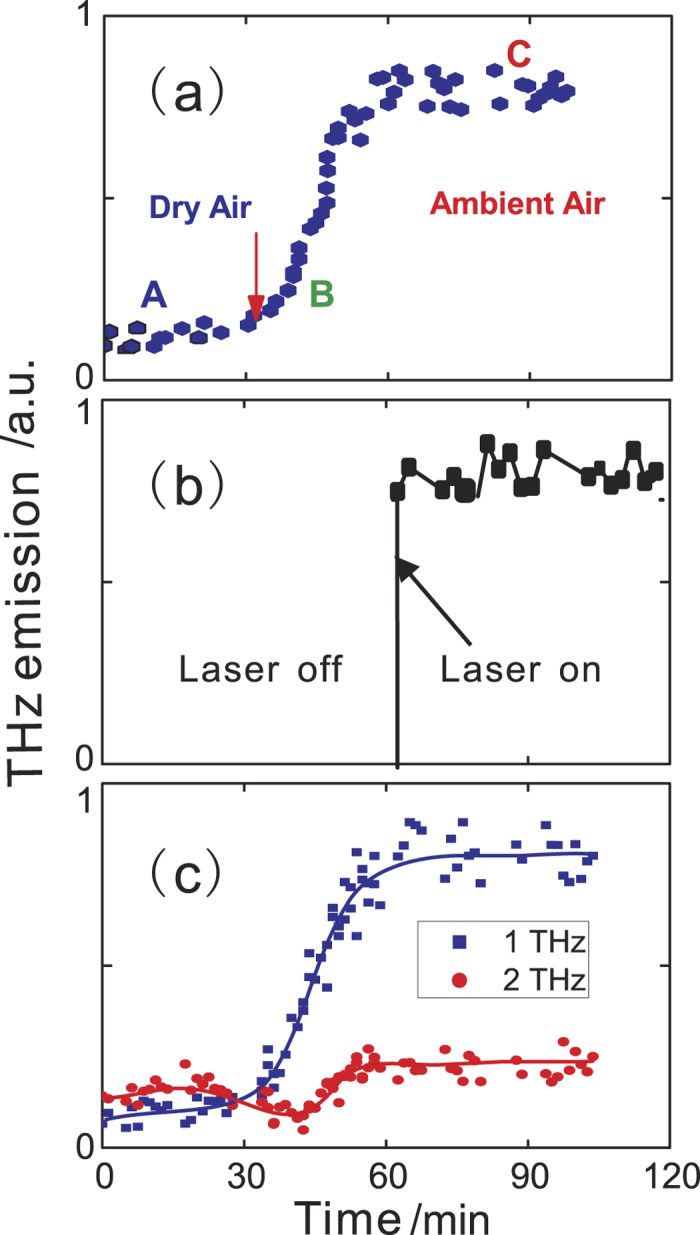
Evolution of the P_in_-P_out_ THz emission from Bi_2_Se_3_ surfaces as a function of time. At time = 0 the sample surface was freshly cleaved and was kept in a dry air purged chamber. Purging was stopped at about 30 min. (**a**) is the evolution of the total emission intensity for the first 2 hrs. A, B and C correspond to the time when the three waveforms of Fig. 4 were recorded. (**b**) is the same as in (**a**) except that the emission was not measured for the first 60 min. and the sample was not exposed to the laser beam. (**c**) is the evolution of the normalized emitted emission at two representative frequencies: 1 THz (blue squares) and 2 THz (red circles). These frequencies were chosen to avoid water vapor absorption lines and to highlight two distinct THz generation mechanisms.

Now we discuss the observed evolution of the THz emission in Bi_2_Se_3_ samples after exposure to ambient air. The observed time dependence of the THz emission suggests that a freshly cleaved surface has a relatively small surface depletion field so that optical rectification dominates the THz emission and a broad bandwidth is observed. Upon exposure to air, a strong depletion field is established on the time scale of ~20 minutes and is maintained at that level afterwards. This effect is not due to exposure to laser irradiation as we show in Fig. 5b by repeating the same sequence without measuring the THz emission, i.e. exposing the sample surfaces to laser irradiation for the first 60 min. The increase of the negative doping density, and consequently, band bending and surface field in Bi_2_Se_3_ from exposure to air has been reported^20^,^27^,^34^,^35^. Current understanding from the ARPES study of the electronic structure as a function of exposure to water vapor^27^,^35^ points to a surface reaction of Bi_2_Se_3_ with water which leads to Se abstraction leaving positively charged vacancies at the surface. Our result that the L_in_-S_out_ emission is largely independent of exposure to air suggests that although the Bi_2_Se_3_ surface is not chemically inert, the surface state remains protected. Our results thus demonstrate that THz emission spectroscopy can potentially provide a valuable spectroscopic tool for studies of the optical conductivity and dynamics of the surface state in centrosymmetric topological insulators such as Be_2_Se_3_. Further studies of THz emission with improved THz detection bandwidth and at varying excitation photon energies (to selectively excite the electronic states) in single crystal Be_2_Se_3_ are warranted.

## Methods

### Samples

Commercial Bi_2_Se_3_ pellets (Alfa Aesar, 99.999% with vacuum deposition grade) of a few mm in size were used in the experiment. They are a highly oriented layered material with surface properties similar to that of the highly oriented pyrolytic graphite (HOPG)^36^. The samples consist of a large number of small crystallites that are stacked perfectly along the <111> direction to form a layered structure. The in-plane orientations of the domains, however, are random which result in isotropic in-plane properties. The material can be easily cleaved with Scotch tape. And aged surface layers were removed to obtain fresh samples for each measurement.

### Experimental setup

THz emission from Bi_2_Se_3_ surfaces was measured with a standard THz time-domain setup based on a femtosecond laser. Details of the setup have been described elsewhere^24^,^36^. In short, the laser source was a Ti:sapphire regenerative amplifier (Spitfire, Spectra Physics) that delivers optical pulses centered around 800 nm and has a ~50 fs pulse duration and 1 kHz repetition rate. The excitation pulse impinges on the sample surface at 45° with a beam diameter of ~1 mm. The incident pump fluence was kept below ~500 μJ/cm^2^, much lower than the sample damage threshold^20^,^26^. A half-wave or quarter-wave plate was used to generate linearly polarized or circularly polarized excitation. Specularly reflected THz radiation (1–10 meV) was collected by a parabolic mirror and focused onto a ZnTe crystal using a second parabolic mirror. The THz electric-field waveform was measured through electro-optical sampling in the ZnTe crystal by varying the time delays between a probe pulse and the THz pulse^24^,^37^. The THz amplitude spectrum was obtained through Fourier transform of the THz electric-field waveform. The s- and p-polarized THz component was selected using a wire-grid polarizer. The setup was placed in a chamber that can be purged with dry air.

## Author Contributions

L.G.Z. and J.S. designed the project; L.G.Z. and B.K. carried out the experiments. L.G.Z., K.F.M. and J.S. wrote the manuscript. All the authors discussed the results and reviewed the manuscript.

## Additional Information

**How to cite this article**: Zhu, L.-G. *et al*. Effect of Surface States on Terahertz Emission from the Bi_2_Se_3_ Surface. *Sci. Rep.* **5**, 10308; doi: 10.1038/srep10308 (2015).
